# Assessment of BDNF serum levels as a diagnostic marker in children with autism spectrum disorder

**DOI:** 10.1038/s41598-020-74239-x

**Published:** 2020-10-15

**Authors:** Alexandre Garcia Barbosa, Riccardo Pratesi, Geysa Stefanne Cutrim Paz, Maria Aparecida Alves Leite dos Santos, Rosa Harumi Uenishi, Eduardo Y. Nakano, Lenora Gandolfi, Claudia B. Pratesi

**Affiliations:** 1 Interdisciplinary Laboratory of Biosciences, School of Medicine, University of Brasilia, Brasilia, DF Brazil; 2 School of Health Sciences, Post-Graduate Program in Health Sciences, University of Brasilia, Brasilia, DF Brazil; 3 Department of Statistics, University of Brasilia, Brasilia, DF 70910-900 Brazil

**Keywords:** Psychiatric disorders, Autism spectrum disorders

## Abstract

There has been a significant increase in autism spectrum disorder (ASD) in the last decades that cannot be exclusively attributed to better diagnosis and an increase in the communication of new cases. Patients with ASD often show dysregulation of proteins associated with synaptic plasticity, notably brain-derived neurotrophic factor (BDNF). The objective of the present study was to analyze BDNF serum concentration levels in children with classic forms autism and a healthy control group to determine if there is a correlation between ASD and BDNF serum levels. Forty-nine children with severe classic form of autism, and 37 healthy children were enrolled in the study. Blood samples, from both patients and controls, were collected and BNDF levels from both groups were analyzed. The average BDNF serum concentration level was statistically higher for children with ASD (P < 0.000) compared to the control group. There is little doubt that BDNF plays a role in the pathophysiology of ASD development and evolution, but its brain levels may fluctuate depending on several known and unknown factors. The critical question is not if BDNF levels can be considered a prognostic or diagnostic marker of ASD, but to determine its role in the onset and progression of this disorder.

## Introduction

Although almost eight decades have elapsed since Leo Kanner^1^ pioneering description of the main characteristics of the autistic disorder, which emphasized the critical deficiency in social interaction and the presence of repetitive and aberrant motor-sensory behavior, its etiology is not yet fully understood. This first original description has hardly changed to the present definition, except for the fact that autism is nowadays regarded as a spectrum with a variable presentation that can range from mild to severe, and therefore the term autism spectrum disorder (ASD) is generally used in this context. However, even in mild cases, most people with ASD require permanent assistance, usually for the rest of their lives^2^. There has been a significant increase in ASD in the last decades that cannot be exclusively attributed to better diagnosis and an increase in the communication of new cases. Consequently, the active search for etiological factors that may explain this increase remains of paramount importance^3^.

The pathophysiology of ASD is complicated and multifactorial, and in most patients, it is not possible to identify any etiological cause for the disorder, despite extensive medical investigations^4^. Several studies have suggested that various genes may be active in the emergence of the behavioral and cognitive abnormalities that characterize ASD. Consequently, although it is reasonable to suppose that both genetic and epigenetic and environmental factors may contribute to the appearance of its clinical phenotype, the etiology of ASD remains elusive^5^.

It has been suggested that synaptic dysfunction may be a possible mechanism for the emergence and progression of postnatal neurodevelopmental disorders^6^. The possibility that the characteristics of the autistic disorder behavior are due to synaptic dysfunction is substantiated by the fact that ASD characteristics are commonly seen in patients with genetic diseases (e.g., fragile X syndrome) where there is a proven interference in synaptic function^7^. Additionally, patients with ASD often show dysregulation of proteins associated with synaptic plasticity, notably brain-derived neurotrophic factor (BDNF)^8,9^. The potential involvement of BDNF in ASD derived from studies on altered BDNF mRNA expression and BDNF protein concentrations in the blood of patients with ASD^8–11^.

BDNF is a member of the neurotrophic family that also includes nerve growth factor (NGF), and neurotrophic factors 3 and 4 (NT3 and NT4)^12^. BDNF participates in a wide range of neurophysiological processes and is present in almost all regions of the brain^13^. The most critical functions of BDNF include the regulation of neurogenesis, glycogenesis, and synaptogenesis, as well as neuroprotection and control of short- and long-duration synaptic interactions that influence memory mechanisms and cognition^14,15^.

BDNF is synthesized in the endoplasmic reticulum as pre-pro-BDNF, transported to the Golgi apparatus, where it cleaves, resulting in the formation of pro-BDNF. This form is cleaved again, resulting in the mature isoform of BDNF (m-BDNF). The pro- to m-BDNF ratio varies among the various stages of the development of the different regions of the brain. During the early postnatal period, higher concentrations of pro-BDNF are found while m-BDNF prevails in adulthood^16^.

Although a close correlation of BDNF levels in serum and central nervous system (CNS) has been widely demonstrated in rats^17^, evidence of this correlation in humans is still lacking. However, it is assumed that peripheral levels of BDNF indirectly reflect the levels of BDNF in the brain^18^. Consequently, the concentration of BDNF in peripheral blood could be considered a potential biological marker in evaluating individuals with ASD. As a result of this assumption, a growing number of articles, reviews and meta-analysis have appeared evaluating the possible changes in BDNF blood levels in ASD^19–22^. However, the results of the studies have been inconsistent, some evidencing reduced BDNF serum^23–27^, while a larger number of other studies have shown elevated BDNF serum levels in children with ASD as compared to healthy controls^8,11,20,28–32^. Additionally, studies evaluating levels of BDNF in neonates who subsequently evolved to an ASD also revealed inconsistent results^33–38^. Therefore, it seems evident that controversies exist regarding both the role of BDNF in the pathophysiology of ASD as to its value as a possible marker of this disorder.

As a consequence of the still existing controversies, the objective of this study was to investigate the serum levels of BDNF in a group of children with severe ASD, comparing them with healthy controls, trying to evaluate the value of BDNF level in serum as a possible auxiliary marker in the diagnosis of ASD.

## Patients and methods

In the present study, we analyzed material from a convenience sample of children with ASD treated at the Child Psychosocial Care Center (Centro de Assistência Psicossocial infantil—CAPSI). CAPSI is a referral center for the Federal District Health Department (SSE-DF), receiving children with behavioral or mental disorders from various hospitals and healthcare centers located in Brasilia and surrounding regions. Generally, when the child is admitted, the diagnosis of ASD has already been made by neurologists or psychiatrists from the pediatric unit of ​​SSE-DF. The diagnosis is subsequently confirmed at the center according to the parameters established in the Diagnostic and Statistical Manual of Mental Disorders—DSM-5^39^.

The study group included children with classic severe forms of ASD. The severity of autistic symptomatology was measured using the Childhood Autism Rating Scale (CARS)^40^. CARS scores range from 15 to 60, and the cutoff point for an autism diagnosis is a score of 30 or above. According to the scoring standards of CARS, scores between 30 and 37 indicate mild to moderate autism and scores between 38 and 60 are characterized as severe autism. All children in the study group had a score equal to or above 37 points.

Children on medication that could in any way interfere with the test result and children with mild, moderate, or atypical forms of ASD such; as Asperger's syndrome, invasive developmental disorders without further specification, Rett's syndrome, fragile X syndrome, and Down's syndrome were excluded from the study.

The control group consisted of children, with no clinical characteristics of ASD, attended at the Central Laboratory of the University Hospital of Brasilia, for routine blood tests (e.g., periodic control exams, acute infectious states, preoperative exams). All parents and guardians, regardless of child's age, signed the consent. Additionally—all children over the age of 12, in addition to having their parents sign, also signed consent. The study was approved by the Health Sciences Teaching and Research Foundation (FEPECS) Ethics Committee of the Federal Secretariat of Health (Protocol # 3,127,531) and followed the guidelines established by the Declaration of Helsinki.

Blood samples, from both patients and controls, were collected in the morning, between 8:00 and 10:00 AM, centrifuged within the first 30 min and the resulting sera were stored at – 80 °C within the following four hours, to avoid possible changes in BNDF levels^25^. All samples were evaluated in duplicate using a commercially available ELISA kit (Biosensis Mature BDNF ELISA Kit, Thebarton, Australia) according to the manufacturer's instructions. The use of this specific product was based on a study by Polacchini et al.^41^ that considered this kit as the one that provided the most reproducible measurements of serum BDNF. The sensitivity of the assay was 2 pg/ml, and the intra and inter-assay coefficients of variation were respectively 4.31% and 6.6%.

### Patient consent

Informed, written, and signed consent was obtained from parents or guardians for children under the age of 12 and from parents, or guardians and children above the age of 12.

### Ethics approval

The study was approved by the Health Sciences Teaching and Research Foundation (FEPECS) Ethics Committee of the Federal Secretariat of Health (Protocol # 3,127,531).

### Statistical analysis

The Shapiro–Wilk normality test was initially applied to verify the distribution of BDNF values in both children with and without ASD. Due to a lack of normality, the Mann–Whitney nonparametric test was applied to highlight a possible significant difference between the ASD and control groups and between the female and male groups. Spearman's nonparametric correlation was performed to identify a possible relationship between age and BDNF levels. Finally, logistic regression was carried out for each group-dependent variable (autistic and controls) to quantify the impact of BDNF levels on the probability likelihood of having ASD.

## Results

The study group consisted of 49 children with classic severe form of autism (44 boys; 5 girls, ages 2–15 years; mean age 6.6 and median age 6). All children enrolled in the study were rated by the Autism Rating Scale (CARS) (Schopler et al.^40^), and displayed a score equal or above 37. The CARS scores range from 15 to 60, and the cutoff point for an autism diagnosis is a score of 30 or above. According to the scoring standards of CARS, scores between 30 and 37 indicate mild to moderate autism and scores between 38 and 60 are characterized as severe autism.

Thirty-seven healthy children (24 boys, 13 girls ages 2–15 years; mean age, 9 and median age 10 years) composed the control group. The characteristics of patients and controls can be seen in Table 1.

**Table 1:** Characteristics of the study subjects.

Variable	Patients (n = 49)	Controls (n = 37)	p
Age (years)	6.74 ± 3.35	9.32 ± 3.54	0.001*
**Sex**			
Male	44 (89.8%)	23 (62.2%)	0.002**
Female	5 (10.2%)	14 (37.8%)	
BDNF (ng/ml)	34.38 ± 2.81	31.24 ± 3.75	0.000*

Frequencies (and percentages) for the categorical variable sex and mean ± SD for the variables age and BDNF levels.

*SD* standard deviation, *BDNF* brain-derived neurotrophic factor.

*Mann–Whitney test.

**Pearson chi-squared test.

The average BDNF serum concentration level was statistically higher for children with ASD (P < 0.000) compared to the control group (34.38 ± 2.81 and 31.24 ± 3.75 ng/ml).

Simple and multiple logistic regression models were applied to establish the diagnosis of ASD tentatively. The covariate's sex and age were considered in employing various regression analysis models. The results are presented in Table 2.

**Table 2:** Results of simple and multiple logistic regression analysis of clinical characteristics that may be associated with ASD.

Variable	Simple logistic regression (crude)			Multiple logistic regression (adjusted)		
	β (SE)	OR* (95% CI)	P	β (SE)	OR* (95% CI)	P
Age (years)	–	–	–	− 0.86 ± 0.088	0.75 (0.63;0.89)	0.001
**Sex**						
Male*	–	–	–	0	1	
Female	–	–		− 1.880 ± 0.706	0.15 (0,04;0.61)	0.008
BDNF (ng/ml)	0.328 ± 0.091	1.39 (1.16;1.66)	0.000	0.396 ± 0.109	1.49 (1.20;1.84)	0.000
Intercept	− 10.564			− 10.084		

*CI* confidence Interval.

*Odds ratio.

**Male is the reference category.

As can be seen in Table 2, a higher BDNF value may be associated with a greater probability of ASD (for both crude and adjusted analysis). Additionally, age had a negative effect on the probability of ASD, that is, for children of the same age and BDNF level, the higher the age, the lower the probability of ASD. Finally, female children were less likely to have ASD.

The results in Table 2, the higher probability of ASD can be calculated using the following formula^42^.

$$Prob\left(ASD\right)=\frac{{e}^{-10.084-0.086AGE-1.880GENDER+0.396BDNF}}{1+{e}^{-10.084-0.086AGE-1.880GENDER+0.396BDNF}},$$

where *GENDER* = 0 if male and *GENDER* = 1 if female.

**Figure 1:**
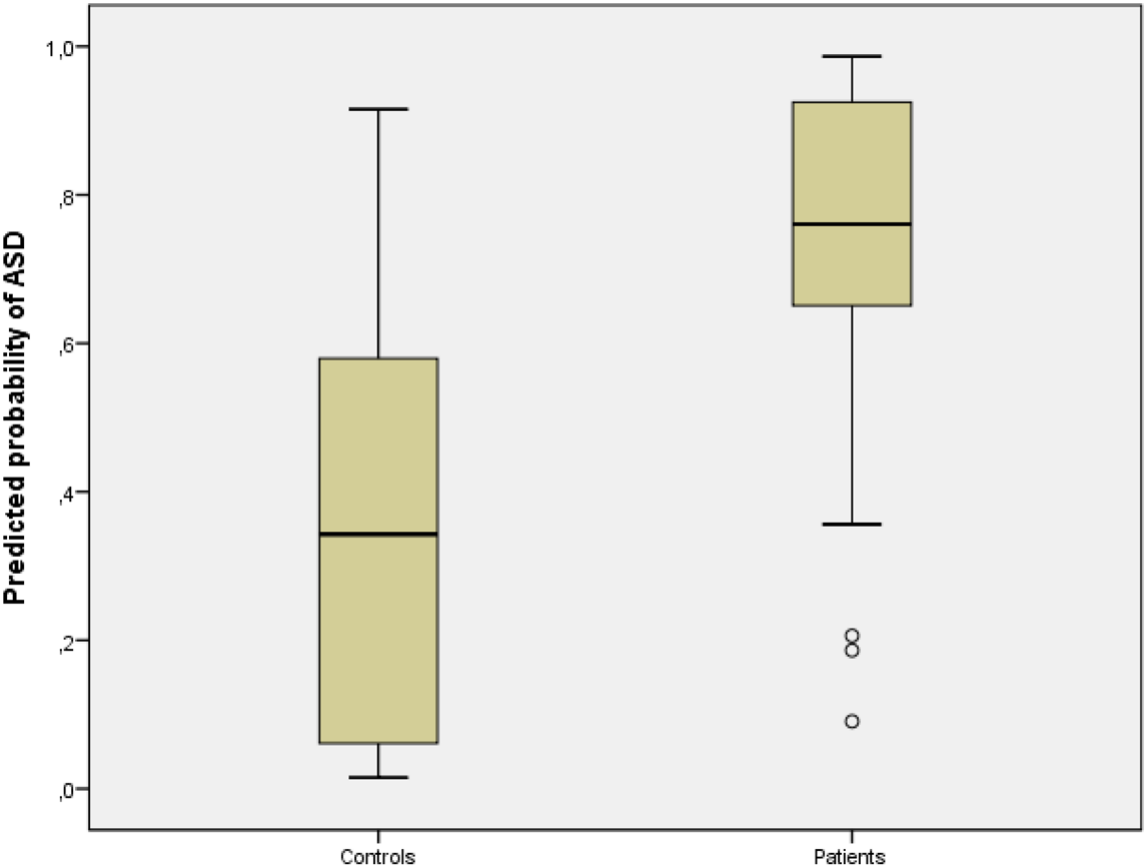
Multiple logistic regression in children with ASD and control cases. The horizontal lines indicate the median value, the box length represents the interquartile range (IQR = Q3–Q1) and the points are the outliers. Despite the statistical significance of the results it is essential to point out that there are clear outliers in both groups.

Figure 1 presents the probability of ASD estimated by multiple logistic regression for the children with ASD and controls cases. In general, children in the study group presented higher level of BDNF (mean: 0.742 ± 0.224) than control group (mean: 0.341 ± 0.276).

A slight correlation between CARS scores and BDNF serum levels was also found, with a Spearman correlation coefficient of 0.070 (P = 0.632). Children of the control group did not undergo CARS evaluation. There is no significant correlation between CARS scores and BDNF serum levels (Spearman correlation coefficient of 0.070, P = 0.632).

## Discussion

In the present study, we found that the median BDNF levels in children with ASD were moderately increased compared to the levels found in healthy children (P < 0.000). However, as seen in Fig. 1, a small number of children, both the study group and the control group, disclosed overlapping BDNF serum levels. These overlapping results agree with most studies on the topic^30,43^ and therefore, significantly influence the sensitivity and specificity of BDNF level as a marker for ASD. These overlapping results significantly influence the sensitivity and specificity of BDNF level as a marker for ASD. However, the use of the BDNF blood levels as an instrument for the diagnosis of ASD has been suggested by several authors^22,24,44,45^.

**Figure 2:**
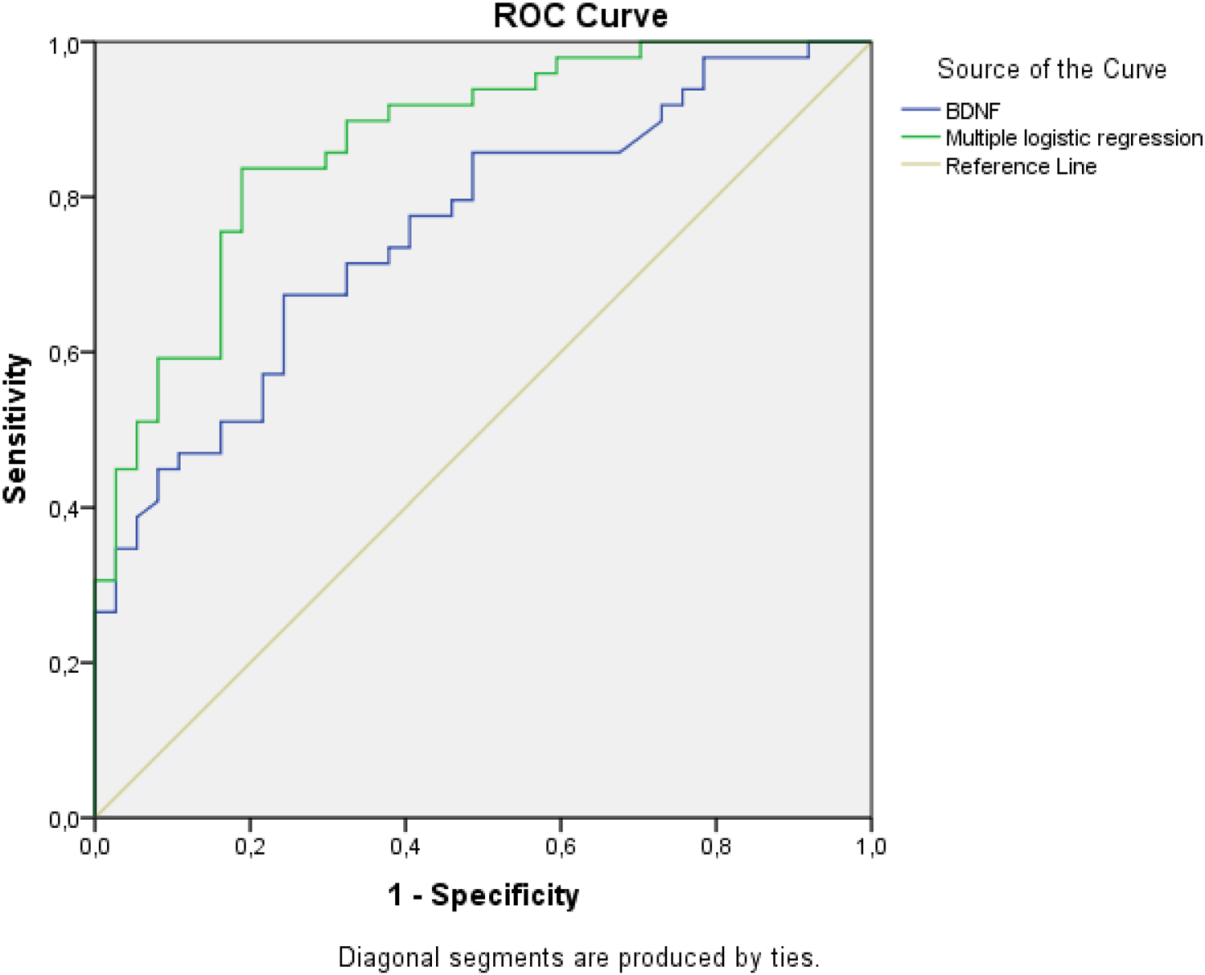
The area under the ROC curve is associated with the discriminating power of a diagnostic test. The values of the ROC curve show the difficulty in discriminating the two groups from the BDNF. The ROC curve for the possible diagnosis of ASD based on serum the BDNF levels and probabilities estimated by multiple logistic regression. In the present configuration the area under the curve (AUC) were 0.758 (95% CI 0.658–0.858) for BDNF and 0.869 (95% CI 0.795–0.944) according to multiple logistic regression.

In our study, according to the results of multiple logistic regression and the receiver operator characteristic (ROC) curve (Fig. 2), the optimal cutoff point (which maximizes the sum of sensitivity and specificity) was 0.60. This cutoff point corresponds to a sensitivity of 83.7% (41/49), a specificity of 81.1% (30/37), and total hits of 82.6% (71/86).

There are many situations where BDNF levels may be altered. Therefore, despite its relatively acceptable sensitivity and specificity, when used as a diagnostic test for ASD, these variables need to be taken into account. Additionally, increased or decreased levels of BDNF have been linked to a variety of disorders. Abnormal BDNF blood levels were described in neurologic and psychiatric diseases, such as schizophrenia^18^, depression and anxiety^46,47^, or even when only depressive personality traits are present^48^. Abnormal levels of BDNF are also detected in genetic syndromes associated with mental retardation and autistic features, such as fragile X syndrome^49^, and Angelman syndrome^50,51^. Serum levels of BDNF are altered in children with intellectual disability and ASD and also in children with other neurodevelopmental disorders, even in the absence of ASD^52^. Other disorders in which abnormalities of BDNF were described are epilepsy^53^, Parkinson disease^54^, and Alzheimer disease^55^. Nutritional quality and physical exercise^56^ have also been linked to altered BDNF levels in patients with ASD. Additionally, BDNF levels may also vary depending on the circadian rhythm^57^, patient’s advancing age^58^, and characteristics of intestinal microbiota^59,60^.

The observation further heightens the doubts regarding the efficacy of BDNF levels as a diagnostic tool for ASD since BDNF levels tend to be higher in children with mental retardation (MR), as observed by Nelson el al.^33^. These authors noted that BDNF concentrations were higher in children with ASD and in those with mental retardation without ASD than in control children. Corroborating their results Miyazaki et al.^61^, determined BDNF levels in a group of adults with ASD and a group of adults with MR. Increased levels were found in both groups, levels being slightly higher among the MR group. Meng et al.^32^, encountered high serum BDNF levels among a group of 82 children with ASD, observing a significant negative association between BDNF serum levels and the children’s low IQ. Furthermore, Bryn et al.^30^ found increased plasma levels of BDNF in children with ASD compared with age- and sex-matched controls, observing that BDNF levels were particularly high in children with intellectual disability. In addition, all ASD patients enrolled in studies whose results revealed normal or low BDNF levels were intellectually normal or had, at least, an IQ over 70^24,27^.

Although controversies regarding BDNF role in ASD still exist, most studies to date point to a variable increase in its blood levels. Consequently, there are no apparent doubts regarding an abnormal functioning of this neurotrophic factor in this disorder. The question is if BDNF is a practical and reliable marker for the diagnosis of ASD. In the face of a disorder with such typical clinical and behavioral characteristics, the confirmation of the diagnosis by means of a marker that may vary depending on the patient’s age^25^, diet, nutritional status and physical activity^56^, circadian rhythm^57,62^, and the characteristics of the intestinal microbiota^59,60^ appears to be of little additional support. Furthermore, despite the scarcity of studies, BDNF levels apparently cannot differentiate ASD from cases of intellectual disability without ASD^52,61^. Additionally, regardless of the inevitable inter and intra-laboratory differences, there is still no systematization of the laboratory technique to be employed in determining BDNF levels^41^. If the determination of its levels had a satisfactory specificity and sensitivity, it would be an important instrument in the identification of newborns who might present ASD, allowing early intervention in these cases, but the few existing studies focusing on this aspect are also controversial^35,37,38^. Our study has a few possible limitations, such as the sample size and the consequent difficulty in obtaining a better normalized cases and controls.

## Conclusion

We feel that the value of BDNF as a marker is relative: if a child displays a classical clinical picture of ASD with normal or decreased blood levels of BDNF, the diagnosis of autism will certainly not be excluded. A review of the studies performed to this date let little doubt that BDNF plays a role in the pathophysiology of ASD development and evolution, but its brain levels may fluctuate depending on several still not wholly known factors. We hope our study highlighted the importance of questioning BDNF levels as a prognostic or diagnostic marker of ASD and highlights the need to understand better the role in the onset and progression of this disorder.

## Data Availability

The statistical analysis from the current study are available from the main researcher on reasonable request. Please allow ten business days for data to be emailed.

## References

[1] Kanner L (1943). Autistic disturbances of affective contact. Nerv Child..

[2] Lord C, Elsabbagh M, Baird G, Veenstra-Vanderweele J (2018). Autism spectrum disorder. Lancet.

[3] Hansen SN, Schendel DE, Parner ET (2005). Explaining the increase in the prevalence of autism spectrum disorders the proportion attributable to changes in reporting practices. JAMA Pediatr..

[4] Rutter M (2005). Aetiology of autism: Findings and questions. J. Intellect. Disabil. Res..

[5] Liu X, Takumi T (2014). Genomic and genetic aspects of autism spectrum disorder. Biochem. Biophys. Res. Commun..

[6] Zoghbi HY (2003). Postnatal neurodevelopmental disorders: Meeting at the synapse?. Science.

[7] Hatton DD, Sideris J, Skinner M, Mankowski J, Bailey DB, Roberts J (2006). Autistic behavior in children with fragile X syndrome: Prevalence, stability, and the impact of FMRP. Am. J. Med. Genet. A..

[8] Miyazaki K, Narita N, Sakuta R, Miyahara T, Naruse H, Okado N (2004). Serum neurotrophin concentrations in autism and mental retardation: A pilot study. Brain Dev..

[9] Nelson KB, Grether JK, Croen LA, Dambrosia JM, Dickens BF, Jelliffe LL (2001). Neuropeptides and neurotrophins in neonatal blood of children with autism or mental retardation. Ann. Neurosci..

[10] Nishimura K, Nakamura K, Anitha A, Yamada K, Tsujii M, Iwayama Y (2007). Genetic analyses of the brain-derived neurotrophic factor (BDNF) gene in autism. Biochem. Biophys. Res. Commun..

[11] Correia CT, Coutinho AM, Sequeira AF, Sousa IG, Lourenço Venda L, Almeida JP (2010). Increased BDNF levels and NTRK2 gene association suggest a disruption of BDNF/TrkB signaling in autism. Genes Brain Behav..

[12] Skaper SD (2012). The neurotrophin family of neurotrophic factors: An overview. Methods Mol. Biol..

[13] Hofer M, Pagliusi SR, Hohn A, Leibrock J, Barde YA (1990). Regional distribution of brain-derived neurotrophic factor mRNA in the adult mouse brain. EMBO J..

[14] Foltran RB, Diaz SL (2016). BDNF isoforms: A round trip ticket between neurogenesis and serotonin?. J. Neurochem..

[15] Kowiański P, Lietzau G, Czuba E, Waśkow M, Steliga A, Moryś J (2018). BDNF: A key factor with multipotent impact on brain signaling and synaptic plasticity. Cell Mol. Neurobiol..

[16] Yang J, Harte-Hargrove LC, Siao C-J, Marinic T, Clarke R, Ma Q (2014). proBDNF negatively regulates neuronal remodeling, synaptic transmission, and synaptic plasticity in hippocampus. Cell Rep..

[17] Karege F, Schwald M, Cisse M (2002). Postnatal developmental profile of brain-derived neurotrophic factor in rat brain and platelets. Neurosci. Lett..

[18] Fernandes BS, Steiner J, Berk M, Molendijk ML, Gonzalez-Pinto A, Turck CW (2015). Peripheral brain-derived neurotrophic factor in schizophrenia and the role of antipsychotics: Meta-analysis and implications. Mol. Psychiatry..

[19] Qin X-Y, Feng J-C, Cao C, Wu H-T, Loh YP, Cheng Y (2016). Association of peripheral blood levels of brain-derived neurotrophic factor with autism spectrum disorder in children: A systematic review and meta-analysis. JAMA Pediatr..

[20] Zheng Z, Zhang L, Zhu T, Huang J, Qu Y, Mu D (2016). Peripheral brain-derived neurotrophic factor in autism spectrum disorder: A systematic review and meta-analysis. Sci. Rep..

[21] Armeanu R, Mokkonen M, Crespi B (2017). Meta-analysis of BDNF levels in autism. Cell Mol. Neurobiol..

[22] Saghazadeh A, Rezaei N (2017). Brain-derived neurotrophic factor levels in autism: A systematic review and meta-analysis. J. Autism Dev. Disord..

[23] Taurines R, Segura M, Schecklmann M, Albantakis L, Grünblatt E, Walitza S (2014). Altered peripheral BDNF mRNA expression and BDNF protein concentrations in blood of children and adolescents with autism spectrum disorder. J. Neural. Transm..

[24] Hashimoto K, Iwata Y, Nakamura K, Tsujii M, Tsuchiya KJ, Sekine Y (2006). Reduced serum levels of brain-derived neurotrophic factor in adult male patients with autism. Prog. Neuropsychopharmacol. Biol. Psychiatry.

[25] Katoh-Semba R, Wakako R, Komori T, Shigemi H, Miyazaki N, Ito H (2007). Age-related changes in BDNF protein levels in human serum: Differences between autism cases and normal controls. Int. J. Dev. Neurosci..

[26] Kasarpalkar, N. J., Kothari, S. T., & Dave, U.P. Brain-derived neurotrophic factor in children with autism spectrum disorder. *Ann. Neurosci*. **21**(2014). https://annalsofneurosciences.org/journal/index.php/annal/article/view/567. 10.5214/ans.0972.7531.210403 PMC4248479 25452672

[27] Francis K, Dougali A, Sideri K, Kroupis C, Vasdekis V, Dima K (2018). Brain-derived neurotrophic factor (BDNF) in children with ASD and their parents: A 3-year follow-up. Acta Psychiatr. Scand..

[28] Connolly AM, Chez M, Streif EM, Keeling RM, Golumbek PT, Kwon JM (2006). Brain-derived neurotrophic factor and autoantibodies to neural antigens in sera of children with autistic spectrum disorders, Landau–Kleffner syndrome, and epilepsy. Biol. Psychiatry.

[29] Ricci S, Businaro R, Ippoliti F, Lo Vasco VR, Massoni F, Onofri E (2013). Altered cytokine and BDNF levels in autism spectrum disorder. Neurotox. Res..

[30] Bryn V, Halvorsen B, Ueland T, Isaksen J, Kolkova K, Ravn K (2015). Brain derived neurotrophic factor (BDNF) and autism spectrum disorders (ASD) in childhood. Eur. J. Paediatr. Neurol..

[31] Wang M, Chen H, Yu T, Cui G, Jiao A, Liang H (2015). Increased serum levels of brain-derived neurotrophic factor in autism spectrum disorder. NeuroReport.

[32] Meng W-D, Sun S-J, Yang J, Chu R-X, Tu W, Liu Q (2017). Elevated serum brain-derived neurotrophic factor (BDNF) but not BDNF gene Val66Met polymorphism is associated with autism spectrum disorders. Mol. Neurobiol..

[33] Nelson KB, Grether JK, Croen LA, Dambrosia JM, Dickens BF, Jelliffe LL (2001). Neuropeptides and neurotrophins in neonatal blood of children with autism or mental retardation. Ann. Neurosci..

[34] Nelson KB (2001). Toward a biology of autism: Possible role of certain neuropeptides and neurotrophins. Clin. Neurosci. Res..

[35] Nelson PG, Kuddo T, Song EY, Dambrosia JM, Kohler S, Satyanarayana G (2006). Selected neurotrophins, neuropeptides, and cytokines: Developmental trajectory and concentrations in neonatal blood of children with autism or Down syndrome. Int. J. Dev. Neurosci..

[36] Croen LA, Goines P, Braunschweig D, Yolken R, Yoshida CK, Grether JK (2008). Brain-derived neurotrophic factor and autism: Maternal and infant peripheral blood levels in the Early Markers for Autism (EMA) study. Autism Res..

[37] Abdallah MW, Mortensen EL, Greaves-Lord K, Larsen N, Bonefeld-Jørgensen EC, Nørgaard-Pedersen B (2013). Neonatal levels of neurotrophic factors and risk of autism spectrum disorders. Acta Psychiatr. Scand..

[38] Skogstrand K, Hagen CM, Borbye-Lorenzen N, Christiansen M, Bybjerg-Grauholm J, Bækvad-Hansen M (2019). Reduced neonatal brain-derived neurotrophic factor is associated with autism spectrum disorders. Transl. Psychiatry.

[39] American Psychiatric Association (2013). Diagnostic and Statistical Manual of Mental Disorders: DSM-5.

[40] Schopler E, Reichler RJ, DeVellis RF, Daly K (1980). Toward objective classification of childhood autism: Childhood Autism Rating Scale (CARS). Kluwer Acad. Publ. Plenum Publ..

[41] Polacchini A, Metelli G, Francavilla R, Baj G, Florean M, Mascaretti LG (2015). A method for reproducible measurements of serum BDNF: Comparison of the performance of six commercial assays. Sci Rep..

[42] Hosmer, D. W., & Lemeshow, S. *Applied Logistic Regression*. Second Edition. Wiley. https://www.researchgate.net/profile/Andrew_Cucchiara/publication/261659875_Applied_Logistic_Regression/links/542c7eff0cf277d58e8c811e/Applied-Logistic-Regression.pdf.

[43] Halepoto DM, Bashir S, Al-Ayadhi L (2014). Possible role of brain-derived neurotrophic factor (BDNF) in autism spectrum disorder: Current status. JCPSP.

[44] Qin X-Y, Feng J-C, Cao C, Wu H-T, Loh YP, Cheng Y (2016). Association of peripheral blood levels of brain-derived neurotrophic factor with autism spectrum disorder in children a systematic review and meta-analysis. JAMA Pediatr..

[45] Zheng Z, Zhang L, Zhu T, Huang J, Qu Y, Mu D (2016). Peripheral brain-derived neurotrophic factor in autism spectrum disorder: A systematic review and meta-analysis. Sci. Rep..

[46] Martinowich K, Manji H, Lu B (2007). New insights into BDNF function in depression and anxiety. Nat. Neurosci..

[47] Sen S, Duman R, Sanacora G (2008). Serum brain-derived neurotrophic factor, depression, and antidepressant medications: Meta-analyses and implications. Biol. Psychiatry.

[48] Lang UE, Hellweg R, Gallinat J (2004). BDNF serum concentrations in healthy volunteers are associated with depression-related personality traits. Neuropsychopharmacology.

[49] Castrén ML, Castrén E (2014). BDNF in fragile X syndrome. Neuropharmacology.

[50] Wink LK, Fitzpatrick S, Shaffer R, Melnyk S, Begtrup AH, Fox E (2015). The neurobehavioral and molecular phenotype of Angelman Syndrome. Am. J. Med. Genet. A..

[51] Peters SU, Horowitz L, Barbieri-Welge R, Taylor JL, Hundley RJ (2012). Longitudinal follow-up of autism spectrum features and sensory behaviors in Angelman syndrome by deletion class: Autism spectrum features in Angelman syndrome. J. Child. Psychol. Psychiatry..

[52] Yeom C-W, Park Y-J, Choi S-W, Bhang S-Y (2016). Association of peripheral BDNF level with cognition, attention and behavior in preschool children. Child Adolesc. Psychiatry Ment. Health.

[53] Iughetti L, Lucaccioni L, Fugetto F, Predieri B, Berardi A, Ferrari F (2018). Brain-derived neurotrophic factor and epilepsy: A systematic review. Neuropeptides.

[54] Scalzo P, Kümmer A, Bretas TL, Cardoso F, Teixeira AL (2010). Serum levels of brain-derived neurotrophic factor correlate with motor impairment in Parkinson’s disease. J. Neurol..

[55] Laske C, Stransky E, Leyhe T, Eschweiler GW, Wittorf A, Richartz E (2006). Stage-dependent BDNF serum concentrations in Alzheimer’s disease. J. Neural. Transm..

[56] Hansen S, Lorentzen J, Pedersen L, Hendrich F, Jorsal M, Pingel J (2019). Suboptimal nutrition and low physical activity are observed together with reduced plasma brain-derived neurotrophic factor (BDNF) concentration in children with severe cerebral palsy. Nutrients.

[57] Liang F-Q, Allen G, Earnest D (2000). Role of brain-derived neurotrophic factor in the circadian regulation of the suprachiasmatic pacemaker by light. J. Neurosci..

[58] Erickson KI, Prakash RS, Voss MW, Chaddock L, Heo S, McLaren M (2010). Brain-derived neurotrophic factor is associated with age-related decline in hippocampal volume. J. Neurosci..

[59] Bercik P, Denou E, Collins J, Jackson W, Lu J, Jury J (2011). The intestinal microbiota affect central levels of brain-derived neurotropic factor and behavior in mice. Gastroenterology.

[60] Sharon G, Cruz NJ, Kang D-W, Gandal MJ, Wang B, Kim Y-M (2019). Human gut microbiota from autism spectrum disorder promote behavioral symptoms in mice. Cell.

[61] Miyazaki K, Naoko N, Sakuta R, Miyahara T, Naruse H, Okado N (2004). Serum neurotrophin concentrations in autism and mental retardation: A pilot study. Brain Dev..

[62] Begliuomini S, Lenzi E, Ninni F, Casarosa E, Merlini S, Pluchino N (2008). Plasma brain-derived neurotrophic factor daily variations in men: Correlation with cortisol circadian rhythm. J. Endocrinol..

